# Global epidemiological insights on the antimicrobial resistance of Enterococcus faecium and a genomic analysis framework

**DOI:** 10.1099/mgen.0.001573

**Published:** 2025-11-20

**Authors:** Kihyun Lee, Yoon-ha Jang, Kuenyoul Park, Heungsup Sung, Mi-Na Kim

**Affiliations:** 1 CJ Bioscience, 14, Sejong-daero, Jung-gu, Seoul, 04527, Republic of Korea; 2 Department of Electrical and Computer Engineering, Texas A&M University, College Station, Texas, 77843, USA; 3 Department of Laboratory Medicine, Sanggye Paik Hospital, Inje University College of Medicine, Seoul 1342, Dongil-ro, Nowon-gu, Seoul 01757, Republic of Korea; 4 Department of Laboratory Medicine, Asan Medical Center, University of Ulsan College of Medicine, 88, Olympic-ro 43-gil, Songpa-gu, Seoul, 05505, Republic of Korea

**Keywords:** antimicrobial resistance, *Enterococcus faecium*, genomic epidemiology, virulence

## Abstract

*Enterococcus faecium* is a pathogen that frequently causes infections and outbreaks in hospitals. Despite the increasing need for genomic surveillance of *E. faecium*, a standardized genomic analysis framework for this pathogen is lacking. We aim to conduct a comprehensive global epidemiological overview and propose a genomic analysis framework. We analysed 21,058 public *E. faecium* genomes from 80 countries to create a comprehensive snapshot of *E. faecium* resistome and virulence in association with geography and population structure. We compared strain clustering based on SNP-based and sequence typing-based approaches with ST80 genomes as examples, to establish the methodological comparability for outbreak investigations using different approaches. Focusing on the resistance genotypes against vancomycin and linezolid, we observed within-sequence type (ST) dynamics, such as a *vanA*-to-*vanB* transition within ST80 isolates in Denmark driven by the clonal expansion and dissemination of mobile linezolid resistance among ST22 and ST32. We established that the popularly used 25 allele distance threshold based on core genome multi-locus sequence typing corresponds to 86 core genome SNPs or 380 whole-genome SNPs. Finally, we introduce *GenoFaecium*, a user-friendly analytical tool to streamline the genomic surveillance of *E. faecium*, which enables consistent interpretation of antimicrobial resistance and virulence profiles from *E. faecium* genome sequences. By providing this tool and empirically bridging the outbreak investigation thresholds of different phylogenetic methods, our study facilitates and improves the genomic surveillance of *E. faecium*.

**Impact Statement**

*Enterococcus faecium* is one of the leading causes of hospital‐acquired infections and presents significant challenges due to the spread of antimicrobial resistance. Although genomic approaches have been utilized in outbreak investigations and resistance prediction of *E. faecium*, a global view of its epidemiology from a genomic perspective has been missing. Specifically, we identify two key gaps: the absence of a practical pathogen‐specific surveillance framework and the lack of comparability across different outbreak‐analysis methods. Here, we analyse publicly available *E. faecium* genomes to determine a global overview of the population structure of *E. faecium* antimicrobial resistance in general and track the regional and temporal dynamics of vancomycin resistance. We introduce an analytic pipeline, *GenoFaecium*, tailored for routine genomic surveillance of this pathogen. Additionally, we empirically evaluate and harmonize clustering thresholds across popularly used genomic methods to provide inter-method comparability. Our findings and tool will have a broad utility to clinical microbiologists and public-health laboratories who seek to implement whole-genome sequencing-based surveillance of *E. faecium* to enhance infection control and track the epidemiology of antimicrobial resistance.

## Data Summary

All genome sequence data analysed in this study were collected from public repositories. The GenBank accession numbers of the genomes and the associated metadata used in this study are presented in the supplementary material. The code developed in this study is available at https://github.com/kihyunee/GenoFaecium.

## Introduction

Vancomycin was approved for clinical use in 1958 and remains a critical treatment option for infections caused by Gram-positive pathogens, including enterococci [1]. First reports of vancomycin resistance in clinical isolates appeared in enterococci during the 1980s [2]. Currently, *Enterococcus faecium*, particularly vancomycin-resistant strains (VREfm), poses a significant threat to healthcare settings and has been designated as a priority pathogen by the World Health Organization [3]. It is a major cause of nosocomial infections [4], including bloodstream, abdominal, urinary tract and medical device-associated infections [5]. The epidemiology of *E. faecium* infections is characterized by the presence of globally disseminated clones, which may reflect adaptation to the nosocomial setting [6]. The contribution of nosocomial transmission to the overall incidence is exceptionally high for VREfm compared to other major bacterial pathogens, as exemplified in a multicentre genomic surveillance study in Australia [7].

Clinical isolates of *E. faecium* often exhibit penicillin resistance due to mutations in penicillin-binding protein 5 (PBP5) genes [8] and high-level aminoglycoside resistance [9], compromising traditional treatment strategies. Vancomycin resistance in hospital-associated *E. faecium* infections is primarily conferred by *van* operons, of which *vanA* and *vanB* variants are the most frequent in clinical isolates [10]. The *vanA* operon is frequently integrated within transposons such as Tn1546, which are commonly associated with plasmids, enabling horizontal gene transfer among *Enterococcus* species in clinical environments [11,12]. In contrast, *vanB* can be found on both plasmids and chromosomes, though chromosomal localization tends to be more prevalent [13]. In addition to vancomycin resistance, the emergence of linezolid resistance is of serious concern in VREfm strains. As linezolid is introduced as a novel antimicrobial for serious VREfm infections, its widespread use led to the rapid emergence of linezolid resistance in VREfm [14]. The resistance is mediated by 23S rRNA mutations [14] or the acquisition of mobile resistance genes such as *cfr*, *optrA* and *poxtA* [15]. Consequently, the emergence of linezolid resistance has recently become a significant concern in the management of VREfm infections [15].

The high frequency of hospital-acquired infections, the existence of epidemic clones, the rapid pace at which clinically relevant antimicrobial resistance emerges and spreads and the involvement of mobile genetic elements in resistance incentivize the monitoring of this pathogen with genomic surveillance methods [16]. Whole-genome sequencing of *E. faecium* isolates can enhance the detection and monitoring of epidemiologically linked cases, newly introduced or expanding clones and emerging or shifting resistance genotypes [17].

Analytical workflows for genomic surveillance of *E. faecium* remain largely unstandardized, which may lead to inconsistent conclusions across studies. Current methods for identifying outbreak clusters primarily rely on various genomic distance metrics, including core genome multi-locus sequence typing (cgMLST), whole-genome SNPs (wgSNPs) and core-genome SNPs (cgSNPs) [18]. Choice of the cutoff value (i.e. distance threshold) critically impacts the sensitivity and specificity of detecting related cases [19,20]. Furthermore, while numerous bioinformatic pipelines exist for profiling antimicrobial resistance and virulence factors from whole-genome sequencing, the results from different pipelines are not consistent, especially for virulence factor profiling [21].

To address challenges in interpreting genomic surveillance data, previous studies have proposed tools as well as frameworks for interpreting sequencing data for pathogens such as *Shigella sonnei*, *Klebsiella pneumoniae* and *Escherichia coli* [22,24]. These studies provided insights from the global genome dataset and strategically proposed the genomic features to be annotated and reported from clinical isolates. To our knowledge, such a study has not been performed on *E. faecium*. This study aims to provide the changing epidemiology of resistance and virulence factors in global *E. faecium* genomes and to provide an annotation tool for use in clinical settings. In addition, we evaluated the concordance of cgMLST, cgSNPs and wgSNPs by comparing them to provide useful baseline data for actual epidemiological investigations of outbreaks.

## Methods

### Antimicrobial resistance data collection

The vancomycin and linezolid resistance rates of *E. faecium* clinical isolates were retrieved from two publicly available sources on 5 March 2024: the SENTRY Antimicrobial Surveillance Program [25] and the One Health Trust’s ResistanceMap (https://resistancemap.onehealthtrust.org/AntibioticResistance.php). Of the retrieved data, we used country-level prevalence of intermediate or resistant strains for downstream comparisons. ResistanceMap data were used for countries with missing SENTRY data. The final data points for vancomycin and linezolid resistance rates spanned 66 and 51 countries, respectively, with 1 to 2,459 surveillance isolates per country.

### Genome data collection

We retrieved 21,058 genome assemblies from the National Center for Biotechnology Information (NCBI) Pathogen Detection database by accessing the entries in the organism group ‘*Enterococcus faecium*’ on 3 March 2024. Only genomes with resolved metadata for the country of origin, collection year and sample source were included. Accession numbers and associated metadata of the genomes are provided in Table S1, available in the online Supplementary Material. We assigned sequence type (ST) numbers to each genome using the mlst tool version 2.23.0, based on the multi-locus sequence typing (MLST) scheme of Homan *et al*. and the allele database provided by PubMLST [26].

### Resistome and virulome analyses

Antimicrobial resistance genes and resistance-conferring point mutations were identified using AMRFinderPlus version 3.12.8 [27] with the default parameters plus the organism specification by ‘-- organism Enterococcus_faecium’. We used a custom script to parse the presence–absence and variant information for the genotypes (i.e. gene family, allele or mutations) associated with resistance to aminoglycosides, beta-lactams, quinolones, macrolides, glycopeptides (vancomycin), oxazolidinones (linezolid), daptomycin and phenicols.

We used a subset of genome assemblies from the NCBI Pathogen Detection database that had associated antimicrobial susceptibility data for vancomycin (*n*=879) or linezolid (*n*=832) to assess the correlation between resistance genotype and phenotype. We calculated the overall agreement, positive predictive agreement and negative predictive agreement between the genotypic and phenotypic classifications using a contingency table. Confidence intervals (95% confidence intervals) were calculated using the Wilson score method without continuity correction.

Genes encoding virulence factors were screened for each genome sequence using ABRicate v1.0.1, in combination with the VFDB release 4 November 2023, with default parameters [28]. We selected virulence genes that were observed in ≥10 genomes and discarded the other low-frequency genes from further analyses. The remaining six virulence genes included microbial surface components that recognize adhesive matrix molecules (MSCRAMMs; *acm*, *fss3*, *ecbA* and *scm*) and biofilm-associated factors (*esp* and *sgrA*).

### Strain typing and phylogenetic analyses

cgMLST allele profiles were assigned using the scheme developed by de Been *et al*. [29] using chewBBACA version 3.3.10. Core-genome SNPs were calculated by first aligning all genome sequences to the reference genome selected for each ST using Minimap2, subsequently determining core conserved loci and SNP variants using a custom script that utilizes Samtools version 1.19 and Bcftools version 1.19. We defined the positions on the reference genome that were aligned by ≥99% of the genomes in the dataset as the core sites and defined the SNPs within these core sites as core SNPs. The whole-genome SNPs were calculated for each pair of genomes using SKA version 0.3.5 [30]. To establish comparable clustering thresholds across phylogenetic methods, we performed a linear regression between cgMLST allele distances and SNP distances (cgSNP and wgSNP) within the ST80 population (*n*=3,456).

To investigate the phylogenetic structure underlying the transition of the *van* genotype in ST80 isolates from Denmark, we reconstructed the maximum likelihood phylogeny of ST80 genomes based on the alignment of cgSNPs using FastTree version 2.1.11, with an outgroup of a non-ST80 genome sequence (GCA_004332055.1). Similarly, we reconstructed cgSNP maximum likelihood trees of the ST32 and ST22 genomes to investigate the phylogenetic distribution of linezolid-resistant genotypes in these STs.

### Development of *GenoFaecium* script

The *GenoFaecium* script was written in Python 3 to provide consistently formatted and epidemiologically relevant annotations of *E. faecium* genome sequences based on observations made in our global genome dataset. *GenoFaecium* is available at https://github.com/kihyunee/GenoFaecium.

## Results

### Geographic distribution of *E. faecium* antimicrobial resistance

**Fig. 1.**
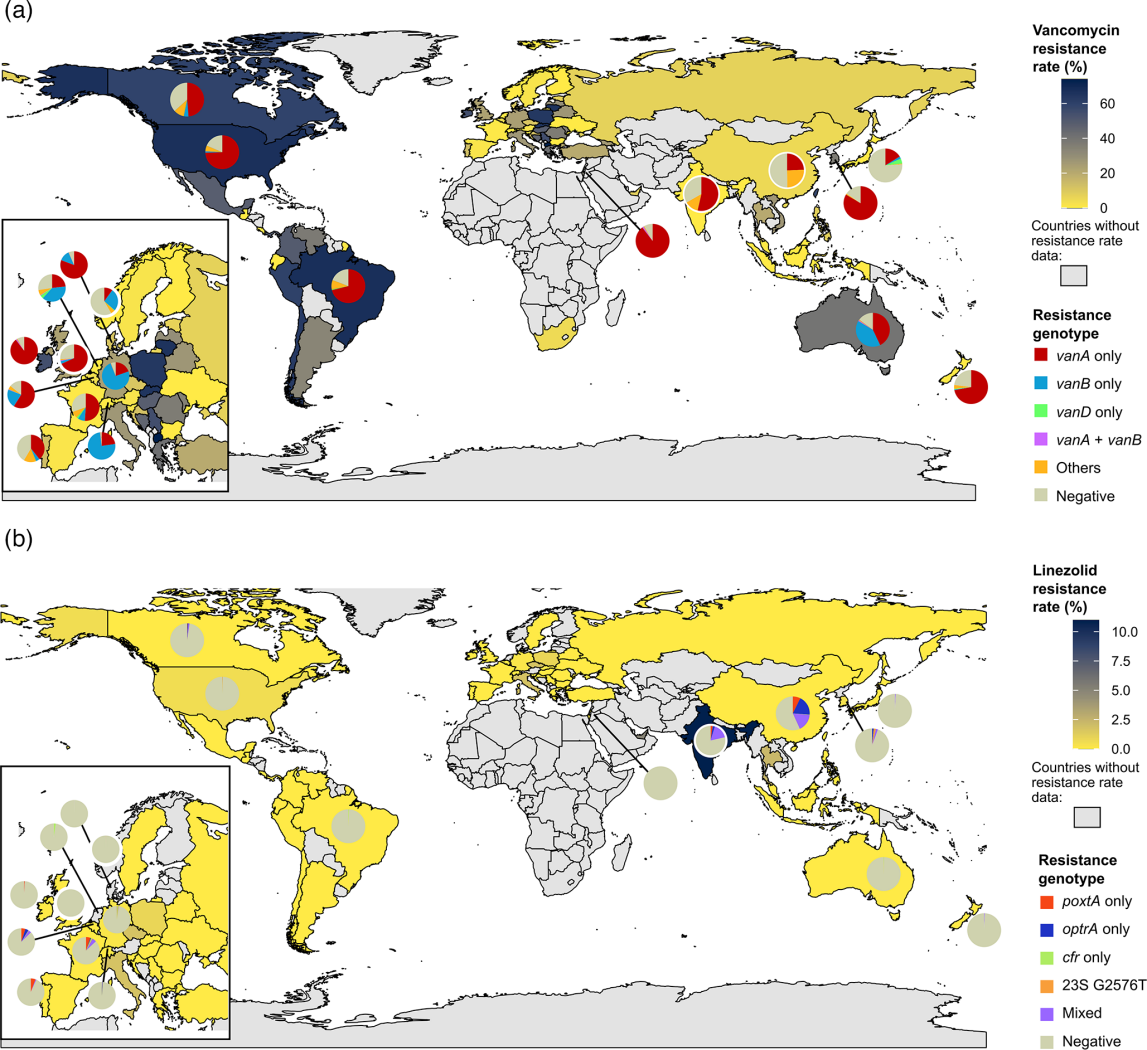
Global map of *E. faecium* resistance rate data collected from SENTRY and One Health Trust databases and the prevalence of resistance genotypes among the *E. faecium* genomes retrieved from the NCBI Pathogen Detection database. (**a**) Map of vancomycin resistance. (**b**) Map of linezolid resistance. Vancomycin resistance rate data were provided for 66 countries by either SENTRY or One Health Trust and 51 countries for linezolid. We treated the remaining countries for which resistance rate data were not available as missing data points and filled them with a grey colour.

The resistance rate data collected from the SENTRY and One Health Trust ResistanceMap represented the prevalence of vancomycin-intermediate or vancomycin-resistant phenotypes among 12,871 *E. faecium* isolates across 66 countries (1–2,459 isolates per country, mean 195) and linezolid-intermediate or -resistant phenotypes among 7,109 isolates across 51 countries (1–2,459 isolates per country, mean 139) (Table S2). Vancomycin resistance rate varied from 0 to 74% across the countries surveyed [median, 21.8%; interquartile range (IQR), 1.2–48.6%] and the linezolid resistance rate from 0 to 11% (median, 0%; IQR, 0–0%) (Fig. 1).

### Global *E. faecium* genome dataset

The collection of global *E. faecium* genomes spanned 769 STs and originated from 80 countries between 1956 and 2024 (Table S1). The prevalence of STs exhibited clear country-specific differences: in the USA, ST17 (14.6%) was the most common, followed by ST736 (11.9%) and ST18 (9.3%); in Denmark, ST80 (28.5%) predominated, with ST1421 (21.8%) and ST203 (20.5%) also prevalent; and in Australia, ST796 (19.5%) was the most frequent, followed by ST1421 (18.2%) and ST203 (12.9%). European countries also showed distinctive patterns, with ST796 predominant in Switzerland, ST117 predominant in Germany and ST80 predominant in the UK. The most globally dispersed clones were STs, ST80, ST17 and ST117, detected in 33–43 countries across 5–6 continents. Some of the highly prevalent clones showed a geographically limited distribution, such as ST796 (*n*=1,034) found only in Australia and Switzerland and ST1471 (*n*=174) in the USA (Table S3).

### Overview of *E. faecium* antimicrobial resistance genes

In the global genome dataset, prevalence of the resistance genotypes was highest for aminoglycosides (99.9%), penicillin (86.6%), quinolones (87.3%) and macrolides (85.0%). The proportion of isolates with resistance genotypes for vancomycin (81.4%) and tetracycline (71.0%) was also high. For the other antibiotics, the overall carriage rates of known resistance genotypes were low: 11.9% for daptomycin, 11.0% for phenicol and 2.0% for oxazolidinone (including linezolid).

Aminoglycoside resistance genotypes were mostly acquired genes such as *aac(6*′)-I (99.8%), *aph(3′)-IIIa* (54.1%) and *ant(6)-Ia* (51.3%). Penicillin resistance was predominantly encoded by point mutations M485A (86.0%) and M485T (0.6%) on PBP5, while beta-lactamases of all types were found in only 0.2% of genomes. Quinolone resistance was also predominantly encoded by mutations, of which *parC* S80I (59.5%) and S80R (24.81%) and *gyrA* S83Y (52.3%) and S83I (27.0%) mutations were dominant. The most frequent vancomycin-resistant genotype was *vanA* (59.8%), followed by *vanB* (18.8%), while the other genotypes showed low frequency (≤0.1%). Regarding daptomycin resistance, we found the co-occurring mutations *liaR* W73C (10.8%) and *liaS* T120A (10.8%) to be dominant genotypes, while mutations in *cls* did not exceed 0.25% of the isolates. Since the analysis relied on assembled contigs, the detection of 23S rRNA mutations, which are the main mechanism of oxazolidinone (linezolid) resistance, was limited. Therefore, most identified resistance determinants were acquired genes, including *optrA* (1.2%), *poxtA* (1.1%) and *cfr* (0.7%).

**Fig. 2.**
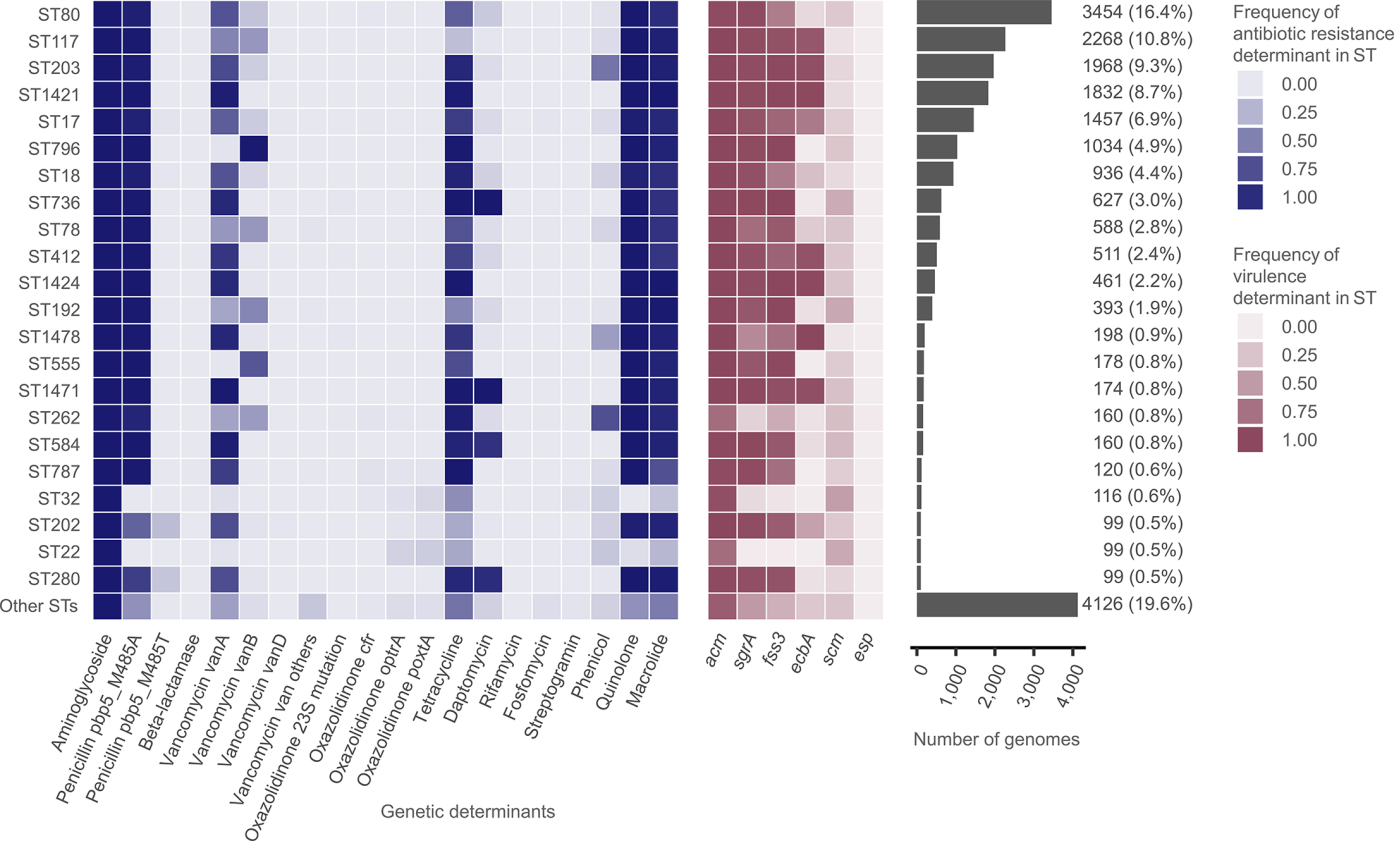
Population structure of the prevalence of genetic determinants for antimicrobial resistance and virulence in *E. faecium*. The heatmap displays the frequency of genetic determinants by STs across the genomic dataset. To focus on the most prevalent lineages, we visualized the top 22 STs based on the number of sequenced genomes in each ST. All remaining 747 less frequent STs were combined into the ‘other STs’ category. The horizontal bar plot on the right side displays the number of genomes analysed per ST.

We observed a clear association between the population structure (i.e. ST) and the distribution of antimicrobial resistance genotypes (Fig. 2). For instance, the PBP5 M485A mutation was observed with a median frequency of 99.3% across STs that had at least 99 genome sequences (STs, *n*=22) but was characteristically absent in ST32 (0/116) and ST22 (0/99). Exceptionally, ST202 and ST280 carried M485T substitution in PBP5 at frequencies of 21.2% and 17.2%, respectively. Daptomycin resistance determinants were found at high frequency in ST1471 (174/174, 100%), ST736 (626/627, 99.8%), ST280 (90/99, 90.9%) and ST584 (141/160, 88.1%), mostly as *liaS* T120A and *liaR* W73C mutations.

### Vancomycin resistance genotypes by geographic location and sequence type

Country-level prevalence of vancomycin-resistant genotypes throughout the genomic surveillance data varied from 23.2 to 98.4% (median, 74.6%; IQR, 59.4–91.7%) when restricted to the 18 countries with at least 100 isolates analysed. Prevalence of the vancomycin-resistant genotype was clearly higher than the resistance rate based on phenotypic surveillance (Fig. 1a) (paired Wilcoxon test, *P*=0.0020; *n*=10 countries where both datasets had ≥100 isolates).

Based on the 879 *E. faecium* isolates with both genomic data and vancomycin categorical phenotypes, the presence of known genotypic determinants was strongly associated with resistant phenotype with positive percent agreement of 97.3% [535/550 phenotype non-susceptible isolates were *van* genotype-positive; 95% confidence interval (CI) 95.6–98.3%], negative percent agreement of 84.5% (278/329 phenotype susceptible isolates were *van* genotype-negative; 95% CI 80.2–88.0%), positive predictive value of 91.3% (535/586 *van* genotype-positive isolates had non-susceptible phenotype; 95% CI 88.7–93.4%) and negative predictive value of 94.9% (278/293 *van* genotype-negative isolates had susceptible phenotype; 95% CI 91.5–97.0%).

**Fig. 3.**
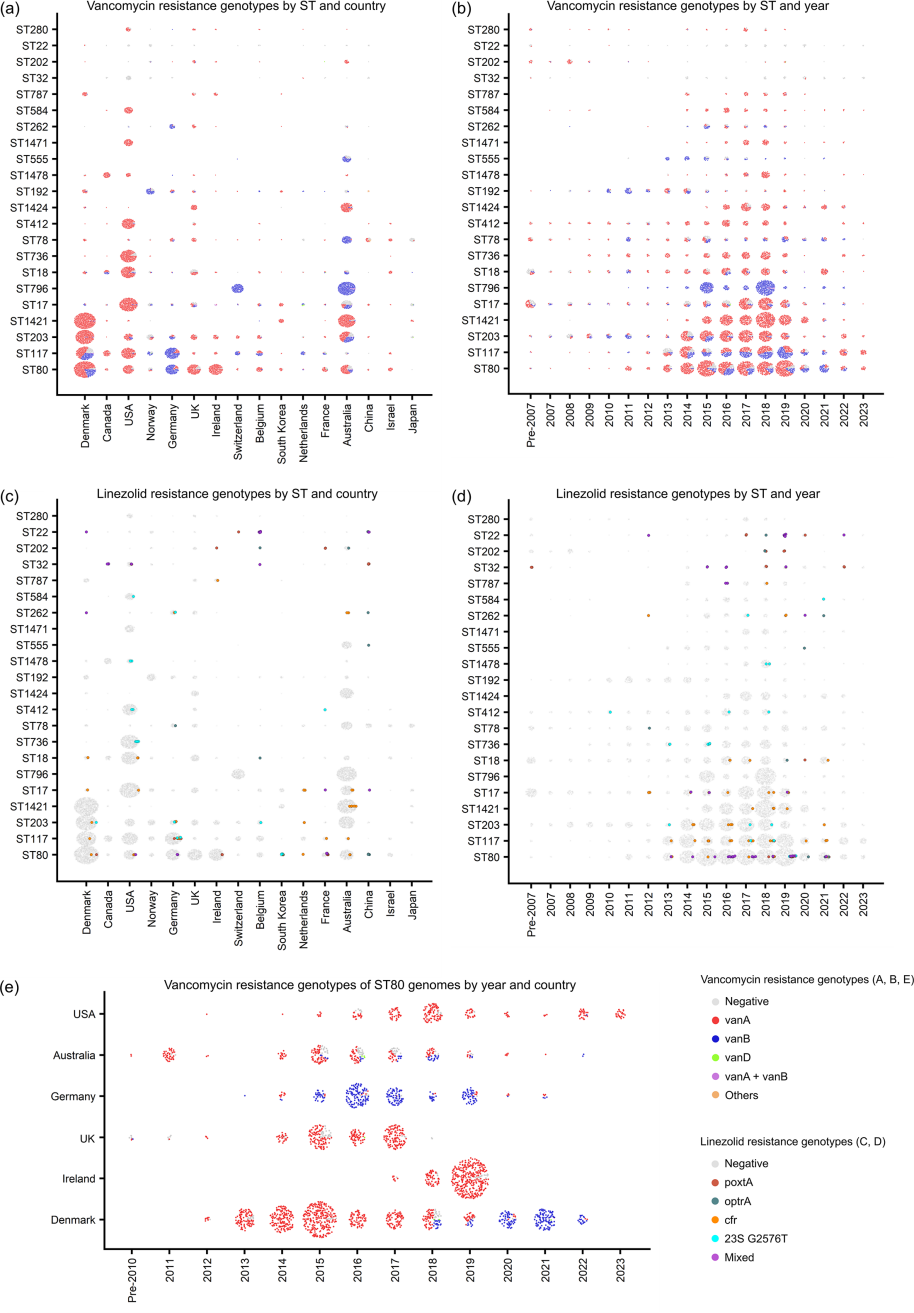
Geographic and temporal distribution of vancomycin and linezolid resistance genotypes in the global *E. faecium* genome dataset stratified by ST. (**a–b**) Vancomycin resistance genotype composition within each ST displayed by country (**a**) and year (**b**). (**c–d**) Linezolid resistance genotype composition within each ST displayed by country (**c**) and year (**d**). (**e**) The distribution of vancomycin resistance genotypes in ST80 genomes from the six countries with large sample sizes, stratified by country and year. In each plot, each dot represents a genome sequence coloured according to the genotype, following the colour code described in the figure key.

**Fig. 4.**
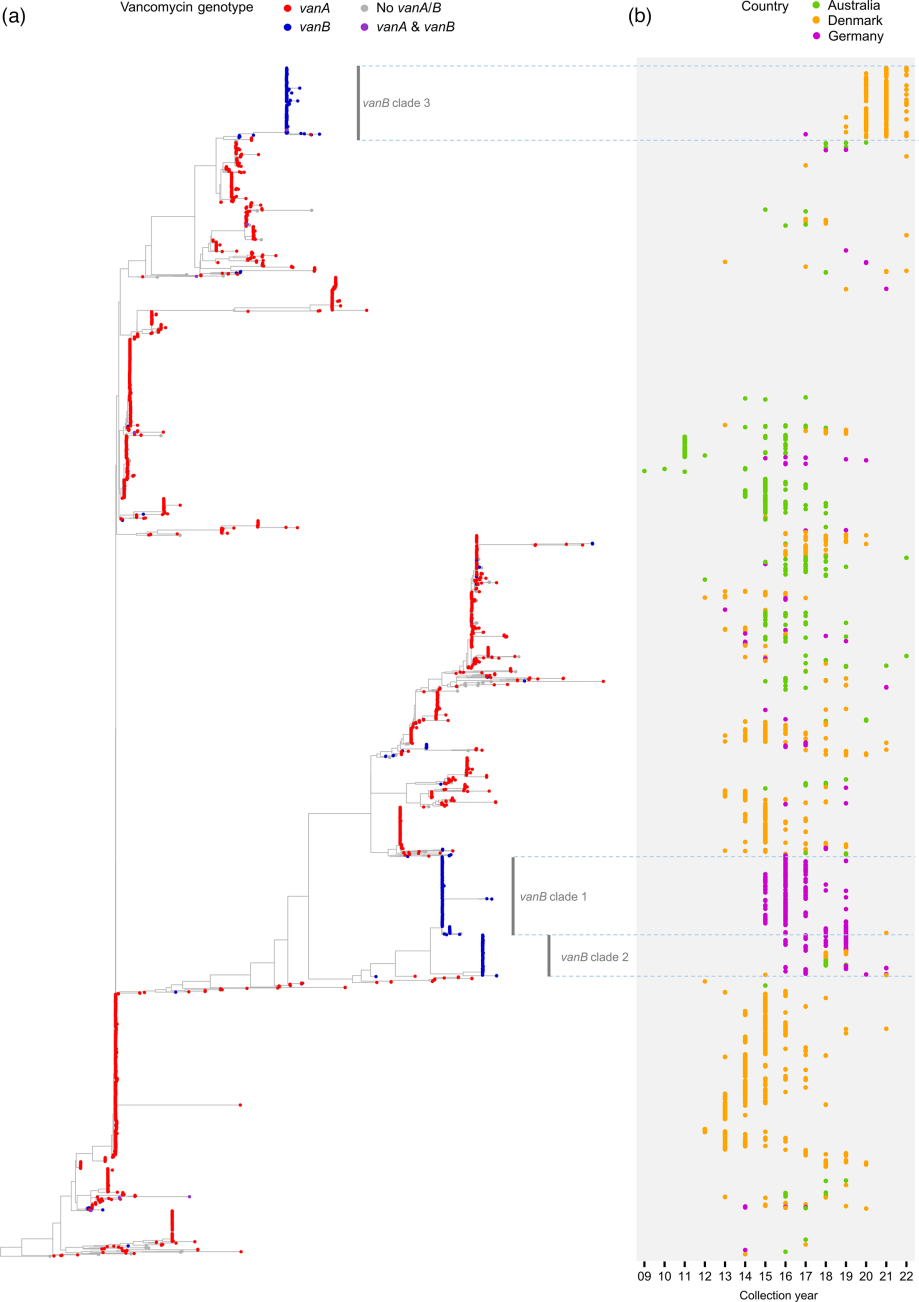
Phylogenetic investigation of *E. faecium* ST80 genomes. (**a**) Maximum likelihood phylogenetic tree of 3,434 ST80 genomes based on 48,988 within-ST80 cgSNP sites. The presence of vancomycin resistance genotype markers in the genomes is displayed by tip colours. (**b**) Collection of years of the *vanB*-positive ST80 isolates from Australia (*n*=46), Germany (*n*=326) and Denmark (*n*=241). Three dominant *vanB*-positive clonal branches were manually identified and highlighted by horizontally dissecting lines and text labels.

The global epidemiology of vancomycin resistance also displayed geographic and temporal dynamics (Fig. 3). Isolates from the USA and Korea predominantly encoded *vanA* (75.2%) rather than *vanB* (1.1%), whereas in Germany, *vanB* (75.2%) was more frequent than *vanA* (19.9%). In Australia, *vanA* (43.9%) and *vanB* (41.6%) were similarly prevalent (Fig. 3a). Inspection of temporal trends in *van* genotype composition revealed a shift in *van* genotype prevalence in some of the major STs, for example, in ST80 (Fig. 3b). We found that in Denmark, the ST80 genomes showed a marked transition from *vanA* to *vanB* around 2018–2019 (Fig. 3e). Phylogenetic relationships among the ST80 genomes from Denmark, Germany and Australia revealed that *vanB* isolates driving this transition in Denmark emerged as a single clone containing pre-transition (2017) Germany isolate (Fig. 4).

### Linezolid resistance genotypes by sequence type

The carriage rate of resistance genotypes ranged from 0 to 43.2% (median, 0.8%; IQR, 0.4–3.3%) in the countries with 100 or more isolate genomes analysed (Fig. 3c, d). The frequency of linezolid resistance genotype was higher than the phenotypic resistance rates of the corresponding countries (Fig. 1b) (paired Wilcoxon test, *P*=0.035; *n*=8 countries where both datasets had≥100 isolates). We observed a poor correlation between the presence of known genotypic markers (i.e. 23S rRNA G2576T mutation, *cfr*, *optrA* and *poxtA*) and the resistance phenotype, with negative predictive value of 89.64% (727/811 genotype-negative isolates were susceptible), positive predictive value of 47.62% (10/21 genotype-positive isolates were non-susceptible), negative percent agreement of 98.51% (727/738 phenotype susceptible isolates were genotype-negative) and positive percent agreement of 10.64% (10/94 phenotype non-susceptible isolates were genotype-positive), based on 832 isolates with both genomic and phenotypic data.

The clones ST32 and ST22, which displayed distinctively low carriage rates of penicillin, quinolone, macrolide, vancomycin and daptomycin resistance genotypes, carried oxazolidinone resistance markers at relatively higher rates (ST32, 9.5%; ST22, 14.1%; vs. 0–3.3% in the other STs, with a median of 0.1%) (Fig. 2). The oxazolidinone resistance markers detected in ST32 and ST22 were acquired genes (i.e. *poxtA*, *optrA* and *cfr* in ST32; *poxtA* and *optrA* in ST22) rather than 23S ribosomal RNA gene mutations. Lineages that acquired linezolid resistance genes tended to be phylogenetically dispersed, rather than clustered within a few branches (Fig. S1).

### Virulence factors of *E. faecium*

Among the genes encoding MSCRAMMs in *E. faecium*, *acm* was consistently prevalent across all major STs (IQR 98.8–100%). In contrast, *fss3* showed varying levels of prevalence among the major STs (IQR 67.5–98.8%), with relatively low prevalence in ST262 (38.8%) and ST80 (67.3%) and >99% prevalence in ST736, ST1424, ST796, ST192 and ST1421. A high inter-ST variation was also observed for *ecbA* (IQR 0.9–65.1%). For biofilm-associated factors, *sgrA* was detected with a high prevalence in almost all major STs (IQR 90.4–97.2%), while *scm* showed a more limited prevalence (IQR 12.2–27.8%) (Fig. 2).

### Comparison of methods for genomic epidemiological investigation of *E. faecium*

**Fig. 5.**
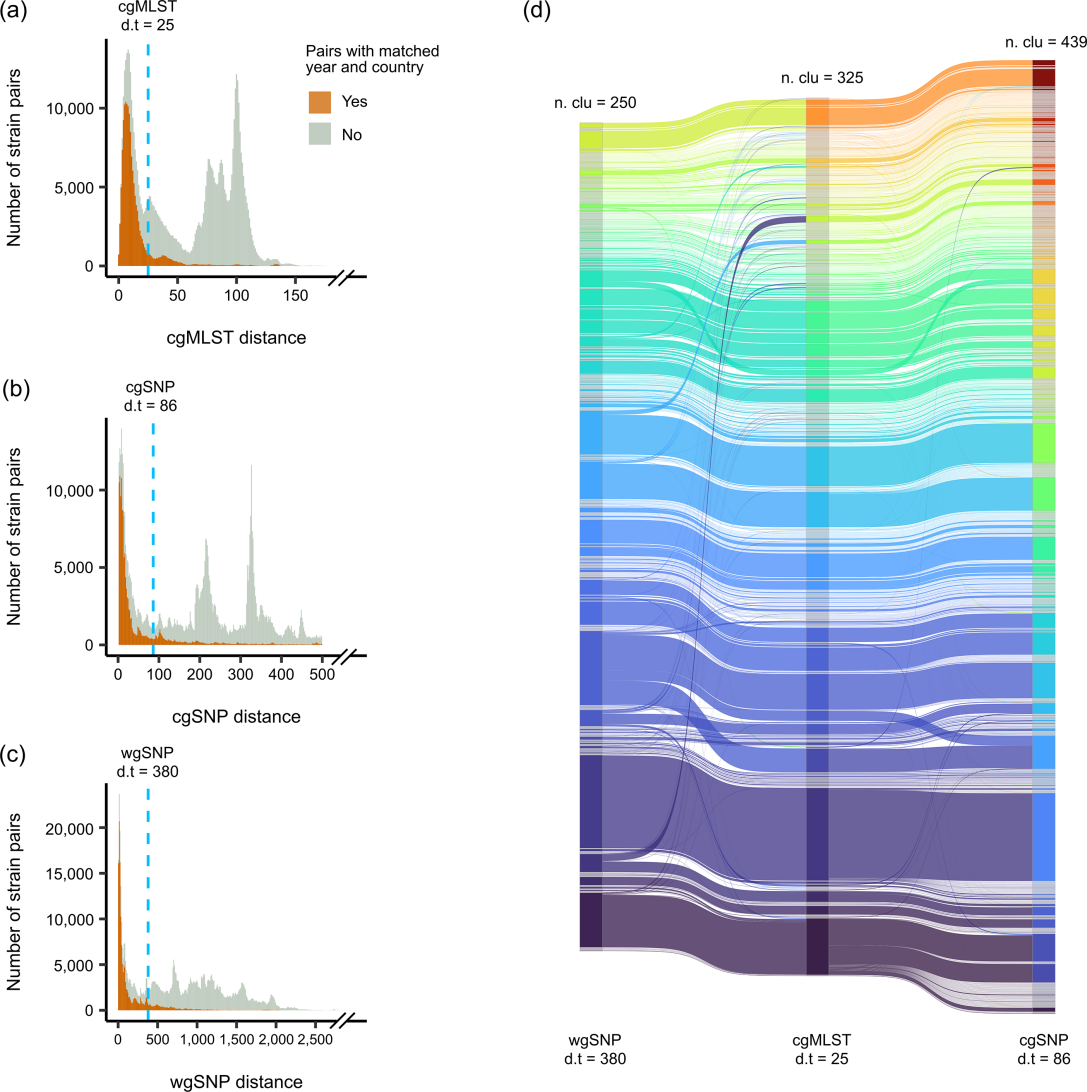
Comparison of genomic epidemiology methods for *E. faecium*. (**a**) Histogram displaying the distribution of cgMLST allele distance throughout pairwise comparisons within ST80. (**b**) Distribution of pairwise cgSNP distance. (**c**) Distribution of pairwise wgSNP distance. In the histograms presented in (**a**)–(**c**), we only included the pairs of genomes that showed a cgSNP distance <500 (*n*=559,556) to enhance the visibility of the distribution at small distance intervals. (**d**) Comparison of clusters generated by cgMLST, cgSNP and wgSNP, with thresholds of 25 alleles, 86 SNPs and 380 SNPs, respectively.

We calculated pairwise genomic distances based on cgMLST, cgSNP and wgSNP methods among the isolates in the ST80 (*n*=3,456), a global clone. All of these three methods captured the presence of closely related isolate pairs, which may be interpreted as possible epidemiological linkages but at different distance scales (Fig. 5a–c). Linear regression analysis indicated that a 25-allele distance in cgMLST corresponded to an 86-SNP distance in cgSNPs and a 380-SNP difference in wgSNPs (Fig. S2). Single-linkage clustering of ST80 genomes based on these distance thresholds (i.e. 25 cgMLST allele distances, 86 cgSNPs and 380 wgSNPs) resulted in 250 clusters for wgSNP, 325 clusters for cgMLST and 439 clusters for cgSNP (Fig. 5d). Under these clustering thresholds, 81.34 and 97.11% of the isolate pairs in the same cgMLST cluster were also clustered together by cgSNP and wgSNP, respectively. Among the isolate pairs in the same cgSNP or wgSNP cluster, 94.85 and 78.99%, respectively, were found in the same cgMLST cluster.

### Integrated toolkit for genomic interrogation of *E. faecium* isolates

**Fig. 6.**
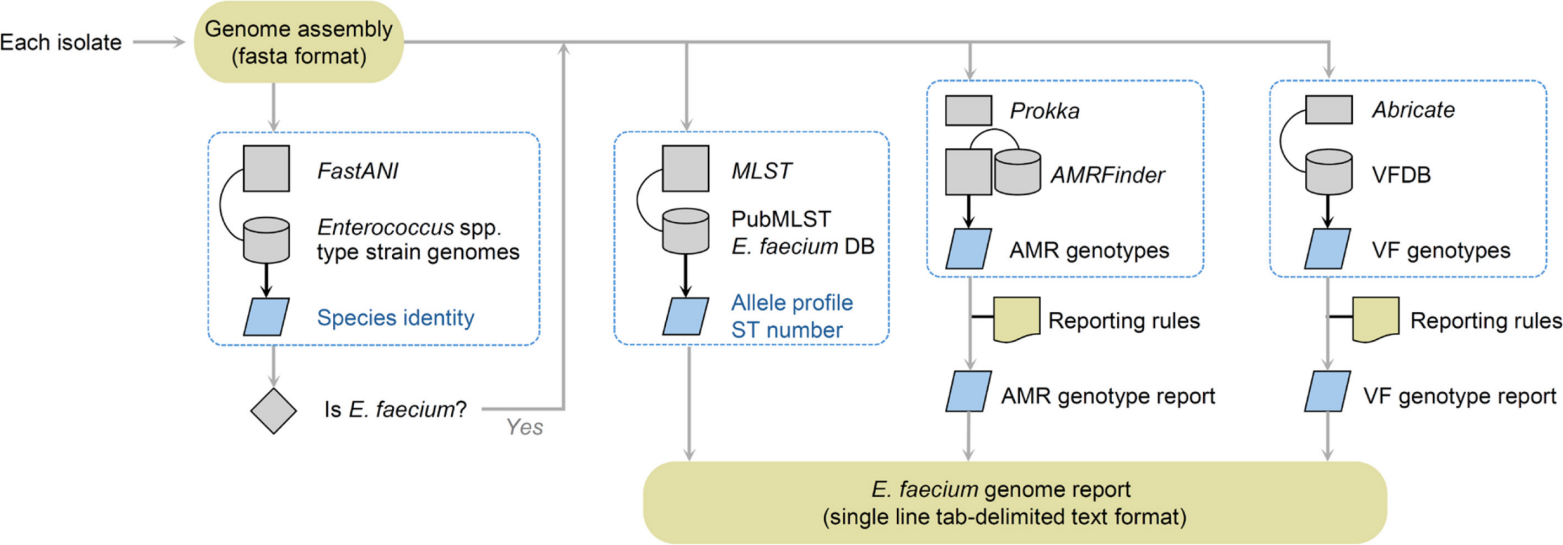
Workflow of *GenoFaecium*, a command-line tool made to simplify the genotypic characterization of *E. faecium* isolates based on whole-genome sequences in clinical and epidemiological investigations.

Based on the assessment of the epidemiology of the resistance and virulence genotypes presented above, we constructed a user-friendly command-line tool called *GenoFaecium* that uses genome assembly files as input and produces a single-line output per input isolate containing epidemiologically relevant features (Fig. 6). For each input genome sequence, *GenoFaecium* confirms species identity, MLST number and allele profile, along with the presence, absence and allele specifications of the curated genotypic markers of *E. faecium* resistance to aminoglycosides, penicillins, vancomycin, oxazolidinone (e.g. linezolid), tetracyclines, daptomycin, rifamycin, fosfomycin, streptogramins, phenicols, quinolones and macrolides. It also provides the presence of genes encoding MSCRAMMs and biofilm-associated factors. Structured outputs from *GenoFaecium* can be easily concatenated across multiple isolates to simplify managing large-scale genomic surveillance data. The database and script of *GenoFaecium* are planned to be updated once every year, but the users can also replace the external databases with the latest versions (e.g. for PubMLST, AMRFinder or VFDB).

## Discussion

In this study, we aimed to extract genomic insights into *E. faecium* epidemiology and develop an accessible genomic analysis tool for this pathogen. Although tools for analysing genes encoding resistance and virulence factors exist, interpreting the results remains challenging without comparison with prior studies on the same pathogen. Therefore, pathogen-specific analytical frameworks have been developed for other bacterial species to facilitate genomic surveillance [22,24]. We investigated the global epidemiology of virulence and antimicrobial resistance of *E. faecium* using the genome dataset and present a streamlined genomic analysis framework for this pathogen to facilitate genomic surveillance of this pathogen. Additionally, we compared three commonly used phylogenetic approaches (i.e. cgMLST, cgSNP and wgSNP) and assessed their correlations.

The geographical distribution of *E. faecium* STs was associated with the regional prevalence and genetic type of vancomycin resistance. Geographic variations in resistance rates and the prevalence of *van* genotypes among countries can be attributed to differences in the dominant clones in each region. In Latin America, studies have reported *vanA*-mediated resistance is primarily driven by two main clones, with ST412 and ST78 being the most common sequence types [31]. In contrast, studies from Sweden identified *vanB*-carrying *E. faecium* belonging to the clonal complex CC17, particularly ST17 and ST125. Similarly, in our study, ST796, a CC17 member, showed the highest proportion of *vanB*.

We also observed increasing *vanB* prevalence in ST80, a sequence type typically associated with *vanA*, in specific countries. One possible explanation for this transition is the spread of *vanB*-encoding plasmids, as previously observed in Australia [32], where a shift from *vanB* to *vanA* within ST80 was driven by plasmid transmission. However, our cgSNP analysis suggests a different mechanism in Denmark, where the *vanA*-to-*vanB* transition observed between 2018 and 2019 was linked to the emergence and clonal expansion of a *vanB*-positive ST80 sublineage. In contrast, our phylogenetic investigation of linezolid resistance within ST32 and ST22 revealed more sporadic phyletic patterns, which indicate multiple independent acquisitions of resistance via mobile genetic elements.

It is worth noting that country-level trends appearing in our analysis (e.g. Fig. 3), especially when a sudden event is observed, could be biassed by over-representation of relatively large datasets from a small number of single hospital-based projects. For example, the increased occurrence of *vanA*-positive ST80 in Ireland was driven by the fact that two published studies contributed all genome sequences that comprised our dataset for Ireland: one focused on 2017–2019 VREfm rectal screening isolates from a hospital in Dublin [33] and another on 2018–2019 VREfm isolates from a different hospital in Dublin [34]. Similarly, while our analysis revealed *vanB*-positive ST796 as the dominant clone in Switzerland (Fig. 3a), the genomic dataset representing Switzerland in our analysis (*n*=485) contains a large proportion of genomes from a single project that investigated a single outbreak by *vanB* VRE of ST796 [35]. The cases from Ireland and Switzerland exemplify current limitations of the dataset available for genomic surveillance and suggest that interpretation of genomic surveillance data should be made carefully, as the results may not reflect the nationwide epidemiology.

Interestingly, the isolates in ST32 and ST22 carried fewer antimicrobial resistance determinants in general, at the same time fewer virulence factors, compared to the globally prevalent STs. Based on a previous report [36] and our data, ST32 and ST22 are frequently isolated from animals and environments. This lack of VFs, combined with their frequent occurrence in non-clinical settings, indicates that these STs represent commensal-like strains. Although clinical isolates in genomic datasets (e.g. those analysed in this study) tend to be enriched in clones known for causing epidemics of hospital-acquired infections, such as ST80, ST117, ST203 and ST1421 [37], enterococci are common colonizers of human gut microbiota [38]. Collectively, this landscape suggests a genomic divergence in *E. faecium* where commensal-like STs (e.g. ST32 and ST22) maintain a limited repertoire of AMR and VF determinants, while the clones prevalent in clinical settings acquire AMR and VF genotypes that optimize adaptiveness in nosocomial environments.

The proportion of isolates carrying antimicrobial resistance determinants was the highest for aminoglycosides and penicillin, whereas resistance determinants for daptomycin and linezolid remained low. This aligned with the phenotypic surveillance data, suggesting that tracking molecular resistance determinants can aid in epidemiological decision-making, especially in anticipation of phenotypic resistance expansion. For instance, plasmid-mediated resistance determinants require multi-species tracking [39]. Hence, the continued tracing of resistance determinants, even for antimicrobials with low resistance rates, is critical.

We observed an overall phenotype-genotype agreement of 92.5% for vancomycin, confirming a strong correlation. As most vancomycin-resistant enterococci outbreaks in human populations are associated with *vanA* and *vanB* [40], this result was expected. Given the strong correlation of *van* genotype presence and phenotype, the discrepancy between the country-level prevalence of resistance phenotype and genotypes likely originates from genomic surveillance bias, such as VRE-focused sampling. The positive predictive value of 91.3% for *van* genes reflects the presence of 51 cases where phenotype was susceptible (‘S’), but the genome sequence had *vanA* (50 cases) or *vanB* (1 case). This type of disagreement may occur biologically through the mutations that alter or silence the expression of *van* operons [41] or antimicrobial susceptibility testing (AST) missing low-level vancomycin resistance [42] and could also be caused by incorrect phenotypes since AST is prone to errors, with variability across methods and laboratories. The positive percent agreement of 97.3% reflects the presence of 15 genomes where we did not find *van* genes, but the phenotype was resistant (‘R’). These genomes came from various STs (i.e. ST80, ST2181, ST78, ST736, ST17, ST2249 and ST412) and countries (the USA, Pakistan, New Zealand and Brazil), suggesting that these isolates are not likely to be carrying a common yet unknown resistance mechanism. It is a limitation of our study that we used the AST phenotype data generated from various sources (i.e. collected from the NCBI Pathogen Detection) and cannot control the impact of the method or protocol used to determine these categorical phenotype data.

In contrast to the previous studies reporting high concordance in detecting linezolid resistance through genotypic methods [43,45], we found the positive percentage agreement for linezolid to be 10.6% and the positive predictive value to be 47.62%. The observed discrepancies can be attributed to several factors. First, the primary resistance mechanism, the 23S rRNA G2576T mutation, is known to have a dosage effect where phenotype correlates with the number of mutant alleles [46]. This non-binary resistance mechanism makes phenotype-genotype correlation challenging, especially considering that short-read genome assembly data will often have minor alleles masked when the mutation sits in multi-copy regions. Furthermore, the presence of acquired resistance genes (*cfr*, *optrA* and *poxtA*) has been reported in phenotypically susceptible *E. faecium* isolates [47], indicating that carriage of these genes does not always confer a resistant phenotype. On the other hand, the presence of isolates with resistant phenotypes that lack any known genotype in our dataset may reflect either the presence of unknown resistance mechanisms or instability in the phenotypic AST data. For instance, in a previous study, re-examination of phenotype for the isolates initially classified as linezolid resistant and genotype-negative turned the result for all such isolates into susceptible [48], suggesting that non-standardized AST methods may contribute substantially to genotype-phenotype disagreement.

Limitations in the prediction of resistance phenotypes (i.e. AST results) solely based on known resistance determinants are a recognized problem. For instance, high rates of incongruence have been reported even in well-studied pathogens such as *E. coli* and *Pseudomonas aeruginosa*, where prediction based on known resistance genes shows accuracy as low as 2% in *P. aeruginosa* and 57% in *E. coli* for certain antibiotics [49]. There are efforts towards the application of whole-genome data and machine learning approaches to capture the combined effects of multiple markers, including regulatory elements and unknown mechanisms, to improve phenotype prediction for certain pathogen–antibiotic combinations [50].

Additionally, we compared three commonly used phylogenetic approaches – cgMLST, cgSNP and wgSNP – and assessed their correlations. The cgMLST approach has been widely adopted in genomic epidemiology studies (Table S4). By providing the corresponding cgSNP and wgSNP cut-offs for commonly used cgMLST thresholds, we enabled bridging the clustering results from one method to another. Our linear regression analyses suggested that 86 cgSNP and 380 wgSNP distances are equivalent to 25 cgMLST allele distances (Fig. S2). Inflated distance cutoff for cgSNP likely reflects the impact of homologous recombination in core genomes, since *Enterococcus* are known to have a high recombination rate [51]. High cutoff value for wgSNP, on the other hand, could be driven by the presence of strain- or lineage-specific accessory genes that increased the size of comparable regions among subsets of strains, often with the genes harbouring higher variability than the core genes.

The main limitations of this study include the limited size of phenotypic data, which hindered correlation analyses between the genotype and clinical resistance profiles, and potential bias toward resistant strains in the genomic dataset, which means population structure seen in this dataset may not reflect that of *E. faecium* in routine clinical settings. The retrospective nature of this dataset may not have captured the current epidemiological landscape. Furthermore, our analysis of antimicrobial resistance genotypes is not likely to encompass the full resistance mechanisms of *E. faecium*, particularly regarding newer antibiotics such as linezolid. These limitations highlight the need for comprehensive phenotypic data, prospective sampling and the continuous updating of resistance databases in future studies.

The global epidemiology of *E. faecium* assessed through genomic datasets, presented in this study, highlights variation in evolutionary mechanisms of the dissemination of antimicrobial resistance in *E. faecium*. This reinforces the importance of continuous globally distributed high-resolution genomic surveillance studies for tracking the dynamics of sequence types and resistance determinants. We propose an analytical framework for *E. faecium* genomic surveillance through a simple and accessible tool, *GenoFaecium*.

## Supplementary material

10.1099/mgen.0.001573 Uncited Supplementary Material 1.

10.1099/mgen.0.001573 Uncited Supplementary Material 2.

10.1099/mgen.0.001573 Uncited Supplementary Material 3.

## Abbreviations

AST: antimicrobial susceptibility testing
cgMLST: core genome multi-locus sequence typing
cgSNPs: core genome SNPs
CI: confidence interval
IQR: interquartile range
MLST: multi-locus sequence typing
NCBI: national center for biotechnology information
PBP5: penicillin-binding protein 5
ST: sequence type
wgSNPs: whole-genome SNP
