# Supplementary material for: Global epidemiological insights on the antimicrobial resistance of Enterococcus faecium and a genomic analysis framework
Source: Microb Genom. 2025 Nov 20;11(11):001573. doi: 10.1099/mgen.0.001573 (PMC12633814; doi:10.1099/mgen.0.001573)
Supplement: Uncited Supplementary Material 1. [file mgen-11-01573-s001.pdf]

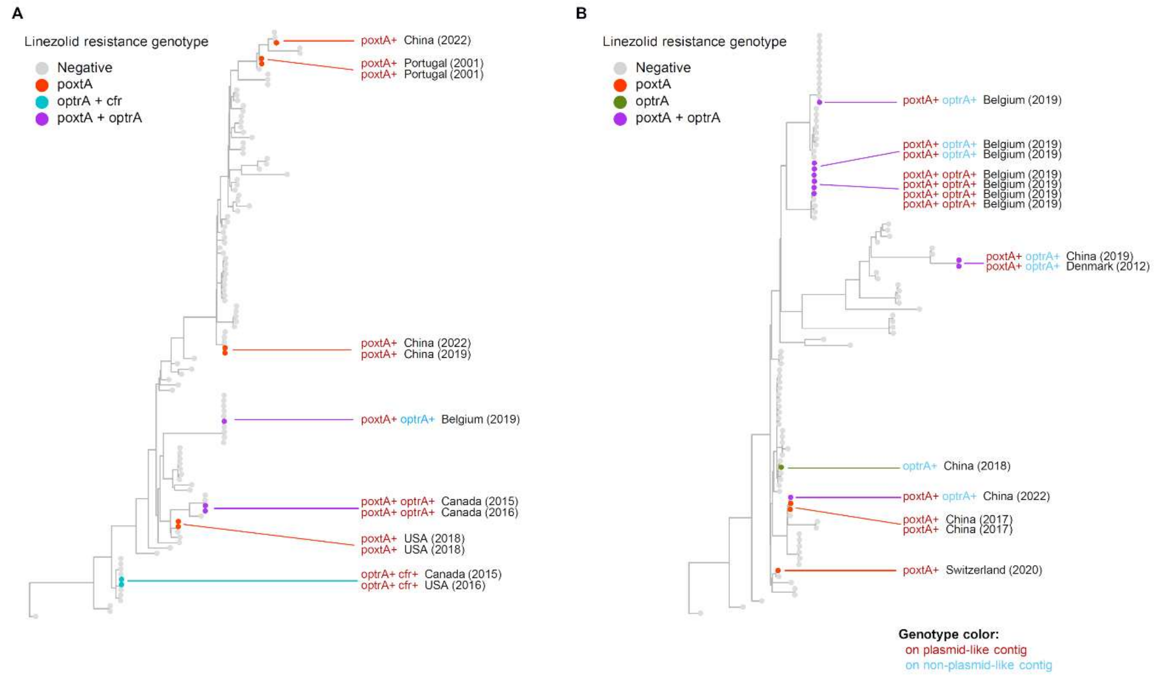

Fig. S1 Phylogenetic investigation of linezolid resistance markers in ST32 and ST22. (A) Maximum likelihood phylogenetic trees of ST32 genomes based on cgSNP sites. (B) Maximum likelihood phylogenetic trees of ST32 genomes based on cgSNP sites. The presence of linezolid resistance markers in each genome (i.e., tree tip) is indicated by tip colors. For the genomes that are positive of linezolid resistance markers, the resistance genotype, country, and year of origin were displayed as text labels.

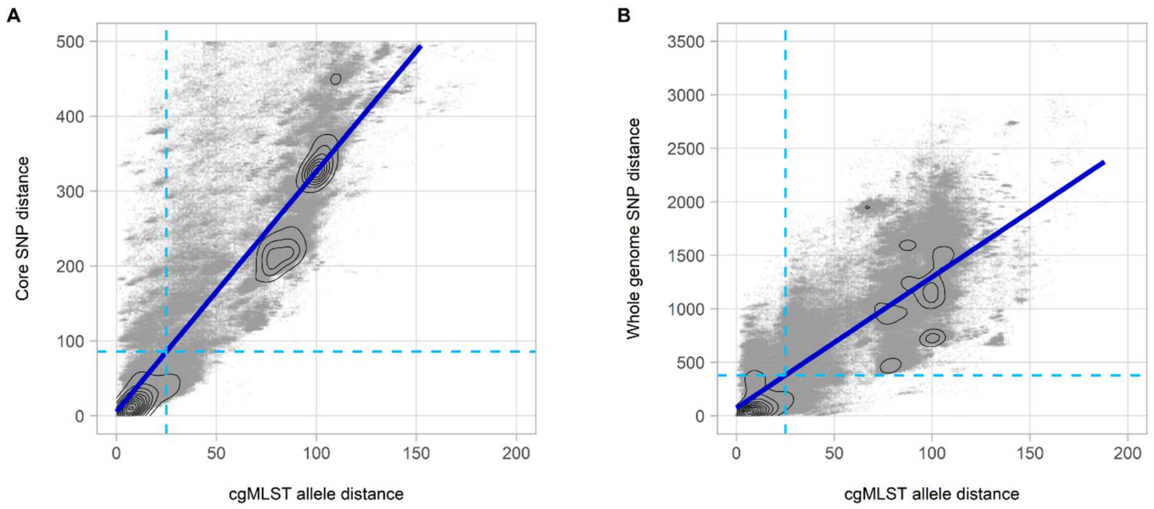

Fig. S2 Relationship between cgMLST allele distance and SNP distance. (A) Scatter plot showing the relationship between cgMLST allele distance and cgSNP distance among the ST80 genomes. (B) Scatter plot showing the relationship between cgMLST allele distance and wgSNP distance among the ST80 genomes. In both panels, only the strain pairs that showed cgSNP <500 were displayed. Contour lines indicate the density of data points. The blue line represents linear regression, and the dotted lines indicate the cgMLST distance of 25 (vertical one) and the corresponding cgSNP or wgSNP distance according to the linear regression.

Supplementary Table S1. Accession numbers and metadata of the 21,058 *E. faecium* genome sequences analyzed in this study. See the supplementary excel file.

Supplementary Table S2. Vancomycin and linezolid resistance rate of *Enterococcus faecium* clinical isolates in various countries around the world according to the phenotypic surveillance data obtained from SENTRY and OneHealthTrust databases.

Vancomycin	One Health Trust		SENTRY		Final data	
Nation	N. isolate	Resistance (%)	N. isolate	Resistance (%)	N. isolate	Resistance (%)
United States of America	1110	68	2459	67.1	2459	67.1
Italy	4736	28	609	26.9	609	26.9
Germany	4721	22	584	24	584	24
Spain	983	1	292	1.4	292	1.4
Turkey	>30	16	264	20.1	264	20.1
South Korea			242	37.2	242	37.2
United Kingdom	>30	23	208	23.1	208	23.1
Ireland	602	28	194	54.6	194	54.6
France	1517	1	164	0.6	164	0.6
Belgium			135	3.7	135	3.7
Greece	950	41	129	41.9	129	41.9
Australia	401	50	115	40	115	40
Poland	900	34	114	64	114	64
Mexico	31	23	109	48.6	109	48.6
Taiwan	298	66	106	58.5	106	58.5
Sweden	984	0	105	1.9	105	1.9
Portugal	409	9	75	13.3	75	13.3
Brazil			71	67.6	71	67.6
Slovenia	219	4	70	1.4	70	1.4
Czech Republic	578	13	55	10.9	55	10.9
Chile			54	63	54	63
Russia	>30	24	54	7.4	54	7.4
Thailand	252	4	44	20.5	44	20.5
Argentina	179	69	42	33.3	42	33.3
China	1398	3	39	5.1	39	5.1
Hungary	710	4	37	48.6	37	48.6
Israel			33	36.4	33	36.4
Canada			32	59.4	32	59.4
Slovakia	219	35	23	65.2	23	65.2
Belarus	>30	5	22	27.3	22	27.3

Romania	191	45	19	36.8	19	36.8
Japan			17	0	17	0
New Zealand			17	0	17	0
Malaysia			15	0	15	0
Philippines			13	7.7	13	7.7
Panama			12	50	12	50
Peru			10	60	10	60
India	314	27	9	0	9	0
Switzerland	>30	2	9	0	9	0
Croatia	113	40	8	12.5	8	12.5
Venezuela	123	51	8	37.5	8	37.5
Bulgaria	148	10	4	0	4	0
Indonesia			4	0	4	0
Costa Rica			3	0	3	0
Colombia			2	50	2	50
Ecuador	30	40	2	0	2	0
Ukraine	>30	7	2	0	2	0
Guatemala			1	0	1	0
Hong Kong			1	0	1	0
Austria	697	2			697	2
Bosnia and Herzegovina	>30	46			31	46
Cyprus	84	51			84	51
Denmark	800	11			800	11
Estonia	83	7			83	7
Finland	261	0			261	0
Georgia	>30	26			31	26
Latvia	113	30			113	30
Lithuania	211	66			211	66
Macedonia, FYR	>30	74			31	74
Moldova	>30	35			31	35
Netherland	1272	0			1272	0
Norway	216	0			216	0
Serbia	>30	55			31	55
South Africa	1945	5			1945	5
United Arab Emirates	346	9			346	9
Vietnam	52	27			52	27
	**One Health Trust**		**SENTRY**		**Final data**	

Linezolid<br>Nation	N. isolate	Resistance (%)	N. isolate	Resistance (%)	N. isolate	Resistance (%)
United States of America			2459	0.6	2459	0.6
Italy			608	1.8	608	1.8
Germany			584	0.2	584	0.2
Spain			292	0	292	0
Turkey	1368	1	264	0	264	0
South Korea			242	0.4	242	0.4
United Kingdom			208	0	208	0
Ireland			194	0	194	0
France			164	0	164	0
Belgium			135	0	135	0
Greece			129	0	129	0
Australia			115	0	115	0
Poland			114	0.9	114	0.9
Mexico			109	0	109	0
Taiwan			106	0	106	0
Sweden			105	0	105	0
Portugal			75	0	75	0
Slovenia			70	0	70	0
Brazil			69	0	69	0
Czech Republic			55	1.8	55	1.8
Chile			54	0	54	0
Russia			54	0	54	0
Thailand			44	2.3	44	2.3
Argentina			42	0	42	0
China			39	0	39	0
Hungary			37	0	37	0
Israel			33	0	33	0
Canada			32	0	32	0
Slovakia			23	0	23	0
Belarus	101	2	22	0	22	0
Romania			19	0	19	0
Japan			17	0	17	0
New Zealand			17	0	17	0
Malaysia			15	0	15	0
Philippines			13	0	13	0
Panama			12	0	12	0

Peru			10	0	10	0
India	36	11	9	0	36	11
Switzerland	224	0	9	0	9	0
Croatia			8	0	8	0
Venezuela			8	0	8	0
Bulgaria			4	0	4	0
Indonesia			4	0	4	0
Costa Rica			3	0	3	0
Colombia			2	0	2	0
Ecuador			2	0	2	0
Ukraine			2	0	2	0
Guatemala			1	0	1	0
Hong Kong			1	0	1	0
Serbia	108	0			108	0
United Arab Emirates	341	5			341	5

Supplementary Table S3. Number of genome sequences counted per sequence type per country of origin. See the supplementary excel file.

Supplementary Table S4. Methods and thresholds used in previously published genomic investigations of *Enterococcus faecium* outbreaks.

Reference		Scope of surveillance/investigation			Scale of surveillance		Outbreak threshold and method
PMID	DOI	Screening subjects	Clinical infections	Environmental	Study scale	Time span	
36748706	10.1099/mgen.0.000937	Yes	Yes	Yes	3 hospitals	7 year	cgMLST 20 alleles
33618041	10.1016/j.jgar.2021.02.007	No	Yes	No	1 hospital	3 year	cgMLST 20 alleles
35082278	10.1038/s41467-022-28156-4	Yes	Yes	No	8 hospitals	15 months	cgMLST 25 alleles
33785076	10.1186/s13073-021-00868-0	-	-	-	Re-analysis of published data		(Plasmid transmission)
38944282	10.1016/j.jhin.2024.06.002	Yes	No	Yes	1 hospital	39 month	cgMLST 20 alleles
35544081	10.1016/S2666-5247(21)00149-X	Yes	Yes	No	4 hospitals	15 months	cgSNP 25 sites
